# Basic life support awareness among medical undergraduate students in Syria, Iraq, and Jordan: a multicenter cross-sectional study

**DOI:** 10.1186/s12245-023-00521-0

**Published:** 2023-07-24

**Authors:** Mohamad Shadi Alkarrash, Mohammad Nour Shashaa, Mohammad Nour Kitaz, Roaa Rhayim, Mohammed Ismail, Sarya Swed, Wael Hafez, M. Ihsan Kaadan, Hamzeh Koumakli, Nour Alhisah, Ahmed Al-Haider, Samer Al-salloum, Ivan Cherrez-Ojeda

**Affiliations:** 1 Faculty of Medicine, University of Aleppo, Aleppo, Syria; 2 Department of Internal Medicine, Medical Research Division, The National Research Centre, Cairo, Egypt; 3 NMC Royal Hospital, 16Th Street, Khalifa City, Abu Dhabi, UAE; 4 Department of Medicine, Boston Medical Center, Boston, MA USA; 5 Department of Medicine, Boston University School of Medicine, Boston, MA USA; 6 Faculty of Medicine, University of Albaath, Homs, Syria; 7 Faculty of Medicine, University of October 6 University, Madaba, Jordan; 8 Faculty of Medicine, University of Al-Qadisiyah, Al-Qadisiyah, Iraq; 9 Emergency Department, Aleppo University Hospital, Aleppo, Syria; 10 Universidad Espíritu Santo, Samborondón, Ecuador; 11 Respiralab Research Group, Guayaquil, Ecuador

**Keywords:** Basic life support, Awareness, Medical students, Cardiopulmonary resuscitation, Syria, Jordan, Iraq

## Abstract

**Background and aims:**

Basic life support (BLS) training rates vary widely worldwide, and there is a general scarcity of surveys that assess students’ knowledge and awareness of BLS in middle eastern nations. This study aims to evaluate medical students’ knowledge and awareness towards basic life support.

**Methods:**

A cross-sectional study, using an online web-based questionnaire, assessing BLS awareness and knowledge, was conducted from 3 to 30 November 2021. The study included 2114 medical students from Syria, Iraq, and Jordan. The questionnaire consisted of three sections: demographic baseline characteristics, knowledge about BLS, and ability to apply basic life support. A binominal logistic regression was done between the total score and other demographic characteristics to determine if we could predict the research sample's appropriate knowledge of BLS level.

**Results:**

There was a moderate knowledge of BLS and cardiopulmonary resuscitation (CPR) skills among participating students with an average score of 19.67 (0–34). Seventy-eight of the participants (1656) stated that they have not attended a basic life support course. There was a significant difference between the participants from different countries, where the mean score in Syria, Jordan, and Iraq was 18.3, 24.3, and 18.8, respectively (*p* < 0.05). Considering the level of knowledge, 18.3%, 72.8%, and 8.9% of the participants had a high, intermediate, and low level, respectively. Furthermore, students who took a BLS course had a higher level of knowledge than those who did not, with an odds ratio of 5.168 (*p* value < 0.0001).

**Conclusion:**

The overall knowledge of medical students’ basic life support knowledge is insufficient and need to be greatly improved. According to this study, previous participation in basic life support training had a positive effect on knowledge level. As a result, universities must establish basic life support programs as quickly as possible.

**Supplementary Information:**

The online version contains supplementary material available at 10.1186/s12245-023-00521-0.

## Background

Cardiac arrest, which happens suddenly, is a significant contributor to mortality on a global scale. A significant proportion of individuals experiencing cardiorespiratory arrest are neonates and infants who have cardiac illnesses [1]. In order to reduce mortality rates associated with severe cardiac and respiratory conditions, it is imperative that healthcare practitioners, including undergraduate medical students during their clinical training, have constant access to the most current emergency protocols. The administration of Basic Life Support (BLS), with particular emphasis on Cardiopulmonary Resuscitation (CPR), is an essential component of cardiac survival [2]. CRP is an emergency critical care technique that aims to maintain adequate breathing and perfusion until the etiology of the cardiac arrest is identified and resolved. Thus, early diagnosis, quick and efficient CPR, and prompt defibrillation are vital for a satisfactory resuscitation result following a cardiac arrest [3]. A trained BLS provider's fundamental skills may be able to lower the fatality rate associated with cardiac arrest in people with cardiovascular disease. Everyone in the community, including medical staff, and students, should be knowledgeable about BLS. Knowledge of BLS should be extended beyond medical staff to the entire public [4, 5].

Regarding CPR knowledge in the medical field, a survey at Riyadh University in Saudi Arabia found that 31% did not have any prior understanding of CPR techniques, and 88% desired to learn CPR [6]. According to another Egyptian survey done in Al-Azhar medical schools, only 27% of students had previously attended BLS courses, and only 34.3% had finished one [7]. Inadequate confidence in performing BLS has also been reported among medical students in Europe [8], and insufficient training among medical students in the U.K., India, Oman, and Iran has also been documented [8–11].

Each country has different BLS training rates according to economics, education level, and war status. In general, low-income Arab countries have inadequate BLS training levels with no clear intention or plans to improve awareness of BLS skills among medical students. Nevertheless, there are not enough observational studies that evaluate BLS knowledge among medical students at preclinical and clinical stages in Arabic countries, especially Jordan, Syria, and Iraq. In Syria, a randomized control trial done by Abbas F et al. found that medical students showed a vast improvement in both practical and theoretical BLS knowledge after taking the BLS course [12]. On top of the previous study, they also found that Jordanian nurses have greatly improved their skills and performance after BLS simulation training [13]. This indicates the elemental importance of BLS courses in improving the skills and apprehension of healthcare workers towards the resuscitation process. Moreover, in a study involving Jordanian adults [14], about a third of participants had received CPR training, and 88.3% would definitely perform CPR on a family member without hesitation. Indeed, low-income Arab countries have the responsibility to provide adequate and accessible training on different levels to health care professionals and the public, as the issue clearly lies in the lack of resources rather than the recipients and course-takers themselves. Teaching the students about BLS principles is essential, especially since the world has faced global pandemics, reporting thousands of deaths in Arab countries daily. Therefore, medical students must be trained to perform this process when required.

Appropriate coordination is a crucial factor in successful CPR; thus, proper practice of the methods and maneuvers is necessary to successfully revive victims, which demands sufficient knowledge and training throughout the medical school years. In order to assess how much benefit medical students will have upon finishing BLS training, we must have data on their current BLS knowledge. Thus, this cross-sectional study intends to assess the knowledge and awareness of medical students from Syria, Jordan, and Iraq towards BLS and CPR skills.

## Methods

### Study design, setting, and data collection

A cross-sectional web-based questionnaire study was conducted from 3 to 30 November 2021, including 6 universities from Syria, 10 from Iraq, and 6 from Jordan. Simple random sampling was utilized to select the included universities. We included all Arab medical students in those universities above 18 years. The data was collected by distributing an online self-administered questionnaire created on Google forms, and announced online via official universities groups, social media, including Whatsapp, Telegram, and Facebook. Participants received all necessary information about the study, including the study’s objectives, their right to withdraw from the study, confidentiality and data protection, and the fact that only fully registered data would be considered for data analysis. After collecting the data, the file was exported to an excel file to safely store the information. Before beginning the data collection, we distributed the questionnaire on 30 participants to evaluate the usability and technical functionality of the online survey. In addition, we assessed the study’s validity using a pilot version of all the items that 20 participants from the target population completed and gave feedback on the questions’ clarity and the survey’s length. These 20 participants were later excluded from the study sample. Cronbach’s alpha scores of 0.72 demonstrated a good internal consistency of the utilized questionnaire. Supervisors and data collectors were instructed on the methods and details of information gathering to maintain data quality. The supervisor and the primary investigator reviewed the accuracy and consistency of the data collection daily. The filled-out information forms were quickly cross-checked with the source data if there seemed to be incompleteness or ambiguity in the recording. Individual entries with missing data were omitted.

### Description of the data collection tool

We designed a closed-questions, electronic questionnaire based on a review of similar published studies [7, 15] and the latest American Heart Association (AHA) guidelines for BLS. Firstly, the questionnaire was written in English. Afterward, we translated it into Arabic. We reported the English version of the study in Additional file 1.

The questionnaire was constituted by three sections as following:

- The first section included demographic variables, such as age, gender, country, residence, university, academic year, academic grade, academic stage (preclinical stage: the first 3 years of the medical education/clinical stage: the last 3 years of the medical education) and attendance in a BLS course previously.
- The second section consisted of 37 questions to assess the participant's knowledge and awareness about BLS (i.e., airway assessment, breathing, CPR technique, AED use, indications of cardiopulmonary resuscitation, signs of successful CPR, suitable responses in emergencies, and dealing with drowning and choking victims). The participants were allocated into three levels according to total score: low (0–12), intermediate (13–24), and high (25–37). One point was attributed to each correct or “yes” responses. Wrong answers or responses of “Don’t know” or “No” did not receive points. The last section consisted of 5 questions about the participants’ opinions of their ability to apply basic life support. We also inquired about the importance of basic life support education in medical colleges' first and second years.

### Sample size

Sample size was calculated using G-power software with the following assumptions: Effect size *f* = proportion of undergraduate medical students from coastal South India (43.8%) (13) and a power (1-B error prob) of 95%. The alpha error prob of 5%.

A sample size of 379 was recommended. The total sample size was 2114, excluding the 26 students who chose not to participate from the 2140 answers we received.

### Statistical analysis

EpiData 3.1 was used to clean, code, and input the data. EpiData 3.1 exported the data to SPSS version 25.0 for further analysis. Data cleaning was done to verify for correctness, consistency, and missing values and variables. Demographic variables, including gender, residence, country, university, academic grade, and academic stage, were described as numbers and percentages. We performed Shapiro–Wilk test to check for data disruption (parametric/non-parametric). Independent-samples *t* test and one-way ANOVA were used to define if there were significant differences in knowledge towards BLS between the subgroups for each demographic variable after confirming the normality and the equal variance assumption. A chi-squared test was used to determine the differences in CRP skills between those who attended a BLS course and those who did not. A binominal logistic regression was conducted between the overall score and other demographic variables to explore if we can predict the adequate knowledge toward BLS level among the study sample depending on the demographic variables. The scale was encoded into two ranges: inadequate knowledge: less than 18 (0 point), and adequate knowledge: 18 or above (1 point). We considered a score above 50% to represent an adequate knowledge toward BLS. For all inferential analysis was considered a significance level of 5%.

### Ethical consideration

Ethical approval was obtained from each University in the three countries to get permission to conduct this research. The Institutional Review Board (IRB) was obtained from Aleppo University/Faculty of Medicine (ALP2195). Participants were received the online survey on the Google form website, which a consent form, based on the most current version of Declaration of Helsinki (64th WMA General Assembly, Fortaleza, Brazil, October 2013) [16], was available on the first page. The next page included relevant information about the study, before the participant starts the study survey.

## Results

### Demographic characteristics of the study sample

A total of 2114 participants from 22 medical universities completed the online questionnaire on the Google form website within 1 month. From those, 1204 were from Syria, 433 were from Jordan, and 477 were from Iraq. Female participants were 55.5% of the sample, and 58.3% were in the preclinical stage. Furthermore, the majority (69.2%) have a moderate academic stage (70–89), but a large majority have not previously attended all did not attend a BLS course (78.3%) (Table 1).

**Table 1:** Demographic characteristics of the participants

Variables	Number	Percent
Total	2114	100%
Gender		
Male	940	44.5%
Female	1174	55.5%
Residence		
City	1638	77.5%
Countryside	476	22.5%
Country		
Syria	1204	57.0%
Jordan	433	20.5%
Iraq	477	22.5%
University		
Syrian universities		
Aleppo University	364	17.3%
Damascus University	218	10.4%
Albaath Univesity	352	16.7%
Tishreen University	145	6.9%
University of Kalamoon	30	1.5%
Hama University	95	4.5%
University of Jordan	66	3.2%
Jordan universities		
Jordan University of Science and Technology	84	3.9%
Hashemite University	100	4.8%
Mutah University	75	3.6%
Yarmouk University	62	2.9%
Al- Balqa' Applied University	46	2.1%
Iraqi universities		
University of Baghdad	59	2.7%
University of Mosul	27	1.2%
University of Babylon	30	1.4%
University of Kufa	43	2%
University of Al-Qadisiyah	45	2.1%
University of Thi-Qar	22	1%
Al Mustansiriyah University	91	4.3%
Ibn Sina University	41	1.9%
University of Kirkuk	64	3%
University of Misan	55	2.6%
Academic stage		
Pre-clinical	1233	58.3%
Clinical	881	41.7%
Academic grade		
60–69	180	8.5%
70–79	745	35.2%
80–89	718	34.0%
90–100	471	22.3%
Have you attended BLS course previously?		
No	1656	78.3%
Yes	458	21.7%

### BLS knowledge

**Fig. 1:**
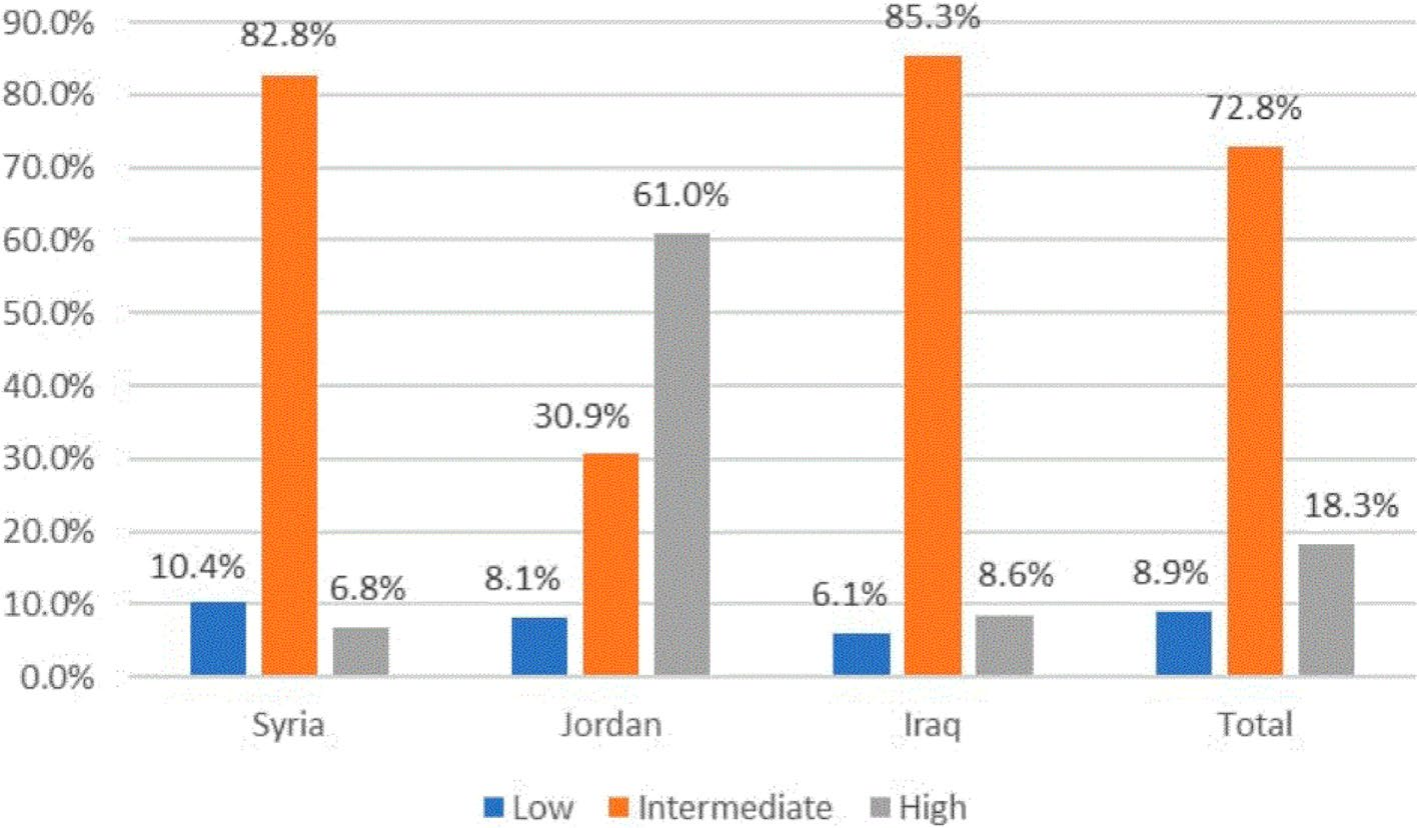
The percentage of the participant according to score’s level

The overall scale score that assesses the knowledge of BLS and CRP skills was 19.67 ± 5.699 (possible maximum score: 37). Figure 1 presents the distribution of participants for the knowledge levels: 18.3%, 72.8%, and 8.9% of participants had high, intermediate, and low knowledge of BLS, respectively. Statistical significant differences (*p* < 0.05) were found between all demographic variables and the BLS knowledge score except for gender. We found that the mean score among males (19.8) was approximately equal among females (19.5). However, there was a noticeable difference among the participants from different countries, where the mean score in Syria, Jordan, and Iraq was 18.3, 24.3, and 18.8, respectively. Furthermore, we encountered different values of the BLS mean score among the participants according to their residence, university, academic grade, and academic stage. Al-Balqa" Applied university in Jordon had the largest mean score (26.4) against Aleppo University in Syria, with the lowest mean score (17.0) of BLS knowledge (Table 2). The participants who attended the BLS course had a higher mean score of 21.6 compared to those who did not attend 19.1 (*p* < 0.05) (Table 2).

**Table 2:** Assessment of the differences between the mean knowledge scores towards BLS and the baseline demographic variables

	Mean	SD	*P* value
Total	19.7	5.7	
Gender			0.205 ^a^
Male	19.8	6.1	
Female	19.5	5.3	
Residence			0.000 ^a^
City	19.4	5.5	
Countryside	20.7	6.2	
Country			0.000^b^
Syria	18.3	4.9	
Jordan	24.3	6.6	
Iraq	18.9	4.5	
University			0.000^b^
University of Aleppo	17.0	5.3	
Damascus University	18.5	4.4	
Albaath University	19.0	4.3	
Tishreen University	18.2	5.5	
University of Kalamoon	18.4	3.7	
Hama University	20.2	3.9	
University of Jordan	21.7	6.6	
Jordan University of Science and Technology	23.6	7.7	
Hashemite University	25.8	5.1	
Mutah University	23.4	6.0	
Yarmouk University	25.0	7.4	
Al- Balqa' Applied University	26.4	6.2	
University of Baghdad	20.8	3.6	
University of Mosul	17.5	3.8	
University of Babylon	18.7	3.7	
University of Kufa	19.9	4.5	
University of Al-Qadisiyah	20.9	4.5	
University of Thi-Qar	21.1	3.6	
Al Mustansiriyah University	17.1	4.1	
Ibn Sina University	17.5	5.1	
University of Kirkuk	19.0	3.7	
University of Misan	18.5	5.3	
Academic stage			0.000^a^
Pre-clinical	18.8	6.3	
Clinical	20.9	4.5	
Academic grade			0.000 ^b^
60–69	17.7	5.0	
70–79	19.6	5.7	
80–89	20.4	5.7	
90–100	19.4	5.7	
Have you attended BLS course previously?			
No	19.1	5.8	0.000^a^
Yes	21.6	4.8	

^a^Independent samples *t* test

^b^One-way ANOVA

We compared the knowledge of CRP skills between the participants who attended BLS training previously and the ones who did not attend a course, in specific relevant domains of CPR, including compression rate, compression location, compression depth, and compression/ventilation ratio. In comparison between those who underwent BLS training and those who did not undergo BLS training significant statistical differences were found between the two groups in all domains (*P* < 0.05), with a higher percentage of participants knowledgeable in CPR skills after attending a BLS course (Table 3).

**Table 3:** CPR skills description among those attended a previous BLS course and those who did not

Variables	Categories	Received training *n* (%)	Did not Receive training *n* (%)	Total *n* (%)	*P* value^a^
Compression rate	Correct	248 (54.1%)	618 (37.3%)	866 (41%)	0.000
	Not correct	210(45.6%)	1038(62.7%)	1248(59%)	
Compression location	Correct	397 (86.7%)	1112 (67.1%)	1509 (71.4%)	0.000
	Not correct	61(13.3%)	544(32.9%)	605(28.6%)	
Compression depth	Correct	293 (64%)	794 (47.9%)	1087 (51.4%)	0.000
	Not Correct	165(36%)	862(52.1%)	1027(48.6%)	
Compression/ventilation ratio	Correct	378 (82.5%)	838 (50.6%)	1216 (57.5%)	0.000
	Not Correct	70(17.5%)	818(49.4%)	898(42.5%)	

^a^Chi-squared

### Perception of the medical students toward BLS

Table 4 shows that a significant percentage of medical students reported to have inadequate skills to perform CRP. For example, only 37.2% agreed or strongly agreed that they were confident in performing CPR. However, 79.1% agreed or strongly agreed that laypeople should learn CPR, and 82.8% agreed or strongly agreed that basic life support education must be applied during the first and second year of the university.

**Table 4:** Perception of the medical students toward BLS

Statement	Frequency		Percent
Am I sure I can perform CPR by myself when required			
	Strongly disagree	245	11.6%
	Disagree	519	24.6%
	Neutral	564	26.7%
	Agree	684	32.4%
	Strongly agree	102	4.8%
Should people outside the medical field be taught CPR?			
	Strongly disagree	55	2.6%
	Disagree	138	6.5%
	Neutral	250	11.8%
	Agree	1092	51.7%
	Strongly agree	579	27.4%
Basic life support education must be applied during the first and second year of the university			
	Strongly disagree	47	2.2%
	Disagree	137	6.5%
	Neutral	179	8.5%
	Agree	1003	47.4%
	Strongly agree	748	35.4%
What is your self-assessment of mastery of Basic Life Support out of 10			
	5 or less	1249	59.1%
	6 or more	865	40.9%
How would you be if you came across someone in need of CPR?			
	Not comfortable	1487	70.3%
	Comfortable	493	23.3%
	Avoid the situation	134	6.3%

### Prediction of adequate knowledge toward of the study sample

Binominal logistic regression model was statistically significant, χ2(8) = 399.31, *p* value < 0. 001. Of the six predictor variables, four were statically significant: country, academic grade and stage, and the attending of a BLS course or not. Students who took a BLS course were 3.54 times more knowledgeable about BLS than other students; moreover, the Jordanian students had a higher level of BLS knowledge than Syrian and Iraqi students, which OR = 5.168(95%CI 3.73–7.14) (Table 5).

**Table 5:** Multivariate logistic regression of predictors for adequate knowledge (score ≥ 18/37) toward BLS among the study sample

Variable	Odds ratio (95%CI)	*P* value
Gender		
Male (reference)	1	
Female	1.058(0.863–1.298)	0.591
Residence		
City (reference)	Reference	
Countryside	1.0360.802–1.33)	0.785
Country		^**< 0.0001**^
Syria (reference)	1	
Jordan	5.168(3.737–7.147)	^**< 0.0001**^
Iraq	1.763(1.305–2.382)	^**< 0.0001**^
Academic stage		
Pre-clinical (reference)	1	
Clinical	4.207(3.344–5.292)	^**<0.0001**^
Academic grade		0.022
60–69 (reference)	1	
70–79	1.605(1.099–2.344)	0.014
80–89	1.831(1.248–2.687)	0.002
90–100	1.600(1.067–2.401)	0.023
Attending BLS course		
No (reference)	1	
Yes	3.540(2.608–4.805)	^**< 0.0001**^

*N* = 2114

## Discussion

The American Heart Association recommends that everyone receive BLS training regardless of their field of study or specialization [17]. In our cross-sectional study, which included 2114 undergraduate medical students from 22 universities in Syria, Jordan, and Iraq, no student was able to respond correctly to all BLS-related questions. Even though several studies have suggested that the medical school system start teaching BLS algorithm, our research found that undergraduate medical students were afraid to apply CPR and had a substantial lack of knowledge on the skills required to perform BLS. Furthermore, 21.7% of the participants stated that they attended BLS courses, which is a result lower than ones reported in Oman (35.2%), [10] Egypt (27%), and [7] Saudi Arabia (22.5%) [18]. Conversely, it was higher than reported in India (18.9%) [15] and Pakistan (14.7%) [19]. Jordan has superior overall results to Syria and Iraq, where 61% of Jordanian students have a high level of education. This can be justified because of the lack of training centers in Syria and Iraq, and due to the internal crisis and the inadequate current economic and educational levels in those countries [20, 21].

The results showed that gender had no apparent effect on knowledge level. In Saudi Arabia, a similar finding was reported [1], although another study in France found a difference between males and females [8]. On the other hand, the results revealed that undergraduate medical students in the clinical stage are more aware of BLS than students at the basic or non-clinical level. Those who have taken prior BLS courses had 1.62 times greater knowledge of BLS than others. Similar results were reported from Oman, [10] the United Kingdom, [19] Pakistan [18]. Although about 72.8% of participants had an intermediate knowledge, most of them (77%) wanted to avoid/be uncomfortable, and about 36.2% were not confident of perform CPR, similarly to the findings that have been reported from India [15] and Europe [22].

When we asked the participants about BLS training, 82.8% of the participants agreed to apply for BLS training during the first and second years of the University. Altintaş et al. also showed that 75.6% of the participants stated that they could confidently apply BLS in real situations after training [23]. According to Pande et al., there is an increase in the mean score of BLS knowledge from 3.42 to 7.42 1 week after the BLS training attendance among first-year medical students [24]. But there is a possibility of knowledge decrease as the time passes after the course. Therefore, students should continually review BLS principles and keep up to date with the latest guidelines.

The findings of a cross-sectional study conducted among health university students in Jordan indicate that the level of cardiopulmonary resuscitation (CPR) knowledge among both trained and untrained individuals was inadequate [25]. However, a separate study that evaluated the perspectives on cardiopulmonary resuscitation (CPR) found that Jordanian students exhibited generally positive and satisfactory attitudes [26]. The findings of our study indicate that the universities in Jordan have attained the top six positions, whereas only one university from Syria has made it to the list of top 10 universities, as presented in Table 5. This serves to corroborate our hypothesis. There is a lack of prior data regarding the knowledge and attitudes of Syrian and Iraqi individuals towards BLS. The Jordanian lead can be attributed to the strong educational system in Jordanian institutions, which has been recognized by the Webometrics Ranking of World Universities as superior to universities in Iraq and Syria [27]. Furthermore, the ongoing regional conflict in Iraq and Syria is likely to have an impact on the educational attainment in the region.

As a reflection on the results of this study, we suggest the following recommendations to enhance the quality and efficacy of BLS principles performed in the three countries, including

- Due to their large audience, we should take advantage of other social media communications such as Facebook and Twitter, as well as television and internet advertisements to realize, emphasize, and enhance the importance of BLS principles in our lives, as seen in a Saudi study that revealed television and movies are the most common sources for improving CPR performance [28].
- Morbidity and mortality can be decreased by providing early training to undergraduate medical students, boosting their confidence in dealing with any emergency or urgent scenario, and performing proper resuscitation.
- We suggest including BLS instruction into the medical curriculum early, followed by ongoing reinforcement via required online examinations.
- To attain this goal, the medical and educational system should be upstaged based on American Heart Association (AHA) principles in the academic curriculum to learn BLS essentials, practical classes and mannequin training are advised.
- To achieve a suitable and healthy environment, international health organizations such as Red Crescent organizations and WHO could provide offer free face-to-face courses for undergraduate medical students, especially at clinical stages.

## Limitations

The major limitation of this study was the data collection technique through an online questionnaire distribution. We did not create any practical examinations to evaluate the pupils' experiences. Another constraint was the heterogeneity of the participants throughout countries, with Syria having the biggest number of students.

## Conclusion

Our findings suggest that medical students' BLS knowledge must be improved. Furthermore, attending a BLS course positively impacted knowledge level, according to this study. As a result, applying for BLS courses before graduation is critical. This procedure will strengthen the educational system’s practical skills and concepts in medical colleges. In general, Jordan had better results than Syria and Iraq, indicating that more recommended adjustments are needed to increase the knowledge among medical students in BLS skills.

## Supplementary Information

**Additional file 1: **Questionnaire. Description of data: data collection instrument in English.

## Data Availability

The data that support the findings of this study are available from the corresponding author upon reasonable request**.**

## Abbreviations

BLS: Basic life support
CPR: Cardiac pulmonary resuscitation
AED: Automated external defibrillation
AHA: American Heart Association
SPSS: Statistical package for social sciences
