# Supplementary material for: Basic life support awareness among medical undergraduate students in Syria, Iraq, and Jordan: a multicenter cross-sectional study
Source: Int J Emerg Med. 2023 Jul 24;16:44. doi: 10.1186/s12245-023-00521-0 (PMC10364399; doi:10.1186/s12245-023-00521-0)
Supplement: Supplementary file 1 — Additional file 1: Questionnaire. Description of data: data collection instrument in English. [file 12245_2023_521_MOESM1_ESM.pdf]

Section 1

1- Sex:
   A) Male
   B) Female

2- Residence:
   A) City
   B) Countryside

3- Country:
   A) Syria
   B) Jordan
   C) Iraq

4- University:
   A) University of Aleppo
   B) Damascus University
   C) Albaath Univesity
   D) Tishreen University
   E) University of Kalamoon
   F) Hama University
   G) University of Jordan
   H) Jordan University of Science and Technology
   I) Hashemite University
   J) Mutah University
   K) Yarmouk University
   L) Al- Balqa' Applied University
   M) University of Baghdad
   N) University of Mosul
   O) University of Babylon
   P) University of Kufa
   Q) University of Al-Qadisiyah
   R) University of Thi-Qar
   S) Al Mustansiriyah University
   T) Ibn Sina University

U) University of Kirkuk
V) University of Misan

5- Academic stage:
A) Pre-clinical
B) Clinical

6- Academic rate:
A) 60 - 69
B) 70 - 79
C) 80 - 89
D) 90 – 100

7- Have you attended a BLS course previously?
A) No
B) Yes

Section 2

1- What is the abbreviation of " BLS "?

A) Begginer life support
B) Basic life supply
C) Basic life support
D) Balanced life support

2- When you find someone unresponsive in the middle of the road, what will be your first response? (Note: You are alone there)

A) try to put him in a safe position
B) calling an ambulance
C) look for safety
D) Running towards him and checking his response

3- If you confirm somebody is not responding to you even after shaking and shouting at him, what will be your immediate action?

A) Try to Re-alert her/him
B) Activate EMS
C) Pulse check
D) Perform a cardiac massage

4- What is the location for chest compression?

A) Left chest
B) Mid Chest
C) Anywhere in the chest
D) Right chest

5- What is the location for chest compression in infants?

A) Mid Chest
B) left chest
C) One finger breadth above the nipple line
D) One finger breadth above the Axillary line

6- If you do not want to give mouth-to-mouth CPR, the following can be done EXCEPT:

A) No CPR
B) Continue performing cardiac massage
C) Putting on a diaphragm that prevents mouths contact and then applying mouth-to mouth resuscitation
D) Ask a paramedic to help you

7- How do you give rescue breathing in infants?

A) only mouth to mouth
B) Mouth-to-mouth and nose
C) Mouth to nose only
A) D)None of the above

8- Depth of compression in children during CPR:

A) 4 or 5 cm
B) 3 or 4 cm
C) 5 or 6 cm
D) 1 or 2 cm

9- Depth of compression in adults during CPR:

A) 5 or 6 cm
B) 3 or 4 cm
C) 6 or 7 cm
D) 8 or 9 cm

10- Depth of compression in neonates during CPR:

A) 4 cm
B) 3 cm
C) 5 cm
D) 6 cm

11- Rate of chest compression in adults and children during CPR:

A) 90/min
B) 100/min
C) 80/min
D) 70/min

12- Ratio of CPR, single rescuer in adults is:

A) 30:2
B) 60:4
C) 90:6
D) 15:1

13- In a new born the chest compression and ventilation ratio is:

A) 3:1
B) 6:2
C) 30:2
D) 60:4

14- What does abbreviation AED stands for?

A) Automated External Defibrillator
B) Acute external defibrillator
C) Acute emergency defibrillator
D) Assistant external defibrillator

15- If you and your friend are having food in a canteen and suddenly your friend starts expressing symptoms of choking, what will be your first response?

A) call an ambulance
B) pulse check
C) Confirm foreign body aspiration by talking to him
D) Perform the Heimlich maneuver immediately

16- You are witnessing an infant who suddenly started choking while he was playing with the toy, you have confirmed that he is unable to cry (or) cough, what will be your first response?

A) put him in the recovery position and talk to him
B) Bcak blows until the foreign body gets out
C) Back blows and chest compression of five cycles, then open the mouth and remove the body only when it is seen
D) Back blows and chest compression of three cycles, then open the mouth and remove the body only when it is seen

17- You are witnessing an adult unresponsive victim who has been submerged in fresh water and just removed from it. He has spontaneous breathing, but he is unresponsive. What is the first step?

A) keep him Prone
B) Performing CPR
C) Give resuscitation breaths until wake up

A) D)keep him in recovery position

18- You noticed that your colleague has suddenly developed slurring of speech and weakness of right upper limb. Which one of the following can be done?

A) Possibly stroke, he may require thrombolysis and hence activate medical emergency services
B) keep talking to him so that he does not lose consciousness
C) Possibly stroke so he should be seated and given water
D) Possibly stroke so he must rest first and hence activate medical emergency services

19- A 50-year-old gentleman with retrosternal chest discomfort, profuse sweating and vomiting. What is next?

A) Probably GERD and there is no need to worry and the patient should be reassured
B) Probably myocardial infarction, hence activate medical emergency service, give an Aspirin tablet and allow him to rest
C) Probably myocardial infarction, give him Nitroglycerin and activate medical emergency service in case of symptoms are failed to improve
D) Probably myocardial infarction, make him lie down

Section 3

indications for cardiopulmonary resuscitation

1- Unconscious person with no palpable pulses and no respiration:

A) Yes
B) No
C) Don't know

2- Victim of road traffic accident with multiple injuries:

A) Yes
B) No
C) Don't know

3- Unresponsive person with normal pulse and respiration:

A) Yes
B) No
C) Don't know

4- Victim of drowning:

A) Yes
B) No
C) Don’t know

5- Burns victim:

A) Yes
B) No
C) Don’t know

6- Choking:

A) Yes
B) No
C) Don’t know

Response to a situation when basic life support is needed

7- Leave the person unnoticed

A) Yes
B) No
C) Don’t know

8- Check the victim for a response

A) Yes
B) No
C) Don’t know

9- Make sure the victim, any bystanders, and you are safe

A) Yes
B) No
C) Don’t know

10- Shout for help

A) Yes
B) No
C) Don’t know

11- Call for an ambulance

A) Yes
B) No

C) Don’t know

12- Do nothing till the help arrives

A) Yes
B) No
C) Don’t know

13- Keep the airway open, look, listen, and feel for normal breathing

A) Yes
B) No
C) Don’t know

14- Give chest compression with rescue breaths

A) Yes
B) No
C) Don’t know

Signs of successful cardiopulmonary resuscitation

15- Spontaneous gasp or breathing

A) Yes
B) No
C) Don’t know

16- Abdominal distention

A) Yes
B) No
C) Don’t know

17- Chest rise and fall with each rescue breathing

A) Yes
B) No
C) Don’t know

18- Return of normal pulse and normal heart beat

A) Yes
B) No
C) Don’t know

Section 4

1- Am I sure I can perform CPR on my own when required

A) Strongly disagree
B) Disagree
C) Neutral
D) Agree
E) Strongly agree

2- Should people outside the medical field be taught CPR?

A) Strongly disagree
B) Disagree
C) Neutral
D) Agree
E) Strongly agree

3- Basic life support education must be applied during the first and second year of the university

A) Strongly disagree
B) Disagree
C) Neutral
D) Agree
E) Strongly agree

4- What is your self-assessment of mastery of Basic Life Support out of ten

A) 5 or less
B) 6 or more

5- How would you be if you came across someone in need of CPR?

A) Not comfortable
B) Comfortable
C) Avoid the situation
